# Acute focal brain damage alters mitochondrial dynamics and autophagy in axotomized neurons

**DOI:** 10.1038/cddis.2014.511

**Published:** 2014-11-27

**Authors:** V Cavallucci, E Bisicchia, M T Cencioni, A Ferri, L Latini, A Nobili, F Biamonte, F Nazio, F Fanelli, S Moreno, M Molinari, M T Viscomi, M D'Amelio

**Affiliations:** 1 Department of Experimental Neurosciences, IRCCS S. Lucia Foundation, Rome, Italy; 2 Institute of Cellular Biology and Neurobiology CNR, Rome, Italy; 3 University Campus Bio-Medico, Rome, Italy; 4 Institute of Histology and Embryology, Catholic University of Sacred Heart, Rome, Italy; 5 Department of Biology-LIME, University ‘Roma Tre', Rome, Italy

## Abstract

Mitochondria are key organelles for the maintenance of life and death of the cell, and their morphology is controlled by continual and balanced fission and fusion dynamics. A balance between these events is mandatory for normal mitochondrial and neuronal function, and emerging evidence indicates that mitochondria undergo extensive fission at an early stage during programmed cell death in several neurodegenerative diseases. A pathway for selective degradation of damaged mitochondria by autophagy, known as mitophagy, has been described, and is of particular importance to sustain neuronal viability. In the present work, we analyzed the effect of autophagy stimulation on mitochondrial function and dynamics in a model of remote degeneration after focal cerebellar lesion. We provided evidence that lesion of a cerebellar hemisphere causes mitochondria depolarization in axotomized precerebellar neurons associated with PTEN-induced putative kinase 1 accumulation and Parkin translocation to mitochondria, block of mitochondrial fusion by Mfn1 degradation, increase of calcineurin activity and dynamin-related protein 1 translocation to mitochondria, and consequent mitochondrial fission. Here we suggest that the observed neuroprotective effect of rapamycin is the result of a dual role: (1) stimulation of autophagy leading to damaged mitochondria removal and (2) enhancement of mitochondria fission to allow their elimination by mitophagy. The involvement of mitochondrial dynamics and mitophagy in brain injury, especially in the context of remote degeneration after acute focal brain damage, has not yet been investigated, and these findings may offer new target for therapeutic intervention to improve functional outcomes following acute brain damage.

Mitochondria are essential organelles for cell function and viability, and are central to several processes such as energy production, metabolism, calcium buffering, and life/death decisions.^1^ Neurons have a high and constant demand for mitochondrial metabolism, to maintain their functions, and contain many mitochondria throughout the cytoplasm, distributed to axons, presynaptic terminals, and dendritic shafts. Mitochondria are highly dynamic organelles that continuously move and change shape. Their morphology is governed by the dynamic equilibrium between fusion and fission processes, both of which are mediated by evolutionarily conserved members of the dynamin family of large GTPases.^2^ Fusion between the outer mitochondrial membranes (OMMs) is mediated by membrane-anchored mitofusins (Mfn1 and Mfn2), whereas that between inner mitochondrial membranes is controlled by optic atrophy 1.^3^ Mitochondrial fission is regulated by dynamin-related protein 1 (Drp1) and fission protein 1 (Fis1).^4^ Drp1 is predominantly expressed in the cytoplasm and is recruited to mitochondria, where it associates with Fis1 to form a complex that constricts the inner and outer membranes, allowing mitochondria to divide.^5, 6^

Mitochondrial dynamics are crucial to the maintenance of mitochondrial function and neuron survival, as evidenced by findings that pathological imbalances between fusion and fission events develop in many neurodegenerative disorders and brain trauma.^7, 8^ Moreover, mitochondrial fission regulates organelle shape and mediates mitochondria-dependent cell death.^9^ The release of proapoptotic factors, such as cytochrome *c* (with consequent formation of the apoptosome and caspase activation), from depolarized mitochondria into the cytosol is a significant event in the induction of apoptosis and is associated with Drp1-mediated fragmentation of the mitochondrial network.^10^

The elimination of dysfunctional mitochondria is therefore a key process with regard to the viability of neurons (and other cell types). Damaged mitochondria that accelerate cell death are removed through autophagy, an evolutionarily conserved lysosome-mediated degradation pathway that maintains the balance between organelle biogenesis, protein synthesis, and degradation of cellular components.^11^

Mitochondria can be selectively degraded by autophagy – a pathway known as mitophagy.^12^ Priming of damaged mitochondria can involve several mechanisms, one of which is triggered by Parkin, a cytosolic E3 ubiquitin ligase that is mutated in familial forms of Parkinson's disease (PD).^13^ Parkin recruitment to impaired mitochondria requires the kinase activity of PINK1 (PTEN-induced putative kinase 1),^14, 15, 16^ a serine/threonine kinase that is also mutated in other autosomal recessive forms of PD.^17^ PINK1 levels are very low in polarized mitochondria, to prevent mitophagy of healthy mitochondria; in contrast, when mitochondria are depolarized, full-length PINK1 accumulates rapidly at damaged organelles, crossing the OMM and acting as cellular sensors of damaged mitochondria.^15^ PINK1 then recruits Parkin to the mitochondrial surface, where it ubiquitinates several OMM proteins, which in turn recruit other proteins to initiate mitophagy.^18^ Mfn1 is a direct substrate of Parkin and its degradation has been suggested to prevent the fusion of damaged and healthy mitochondria.^19^ Drp1-dependent fission of mitochondria is also a crucial event that effects their degradation through mitophagy and the inhibition of fission specifically prevents mitochondrial autophagy.^20^

In this study, we examined mitochondrial function and its relationship with autophagy machinery in an *in vivo* model of acute focal CNS (central nervous system) lesion, focusing on remote changes that are induced by hemicerebellectomy (HCb). Unilateral HCb is a suitable model in which axonal damage-induced neuronal death mechanisms can be studied.^21, 22^ In this model, neuronal degeneration is caused by target deprivation and axonal damage of contralateral precerebellar nuclei of the inferior olive and pontine nuclei. Remote damage is a multifactorial phenomenon in which many components become active in specific time frames^21^ and is significant in determining the overall clinical outcome in many CNS pathologies, including spinal cord injury and traumatic brain injury.^23, 24, 25^

Recently, we demonstrated that autophagy is activated in axotomized neurons after HCb, subsequent to cytochrome *c* release from mitochondria.^26^ Further, rapamycin-enhanced autophagy has neuroprotective effects, reducing neuronal death and improving functional recovery. In this study, we examined mitochondrial dynamics and function in axotomized neurons after HCb in mice and analysed the effects of rapamycin on selective elimination of damaged mitochondria.

## Results

### HCb causes mitochondrial dysfunction and fragmentation in axotomized neurons

Remote degeneration after HCb is primarily an apoptotic process in which death signals, transported retrogradely to axotomized neurons, damage mitochondria and ultimately lead to DNA fragmentation and cell death.^27, 28^ In this scenario, autophagy activation in the early stages of neuronal damage has been interpreted as a reactive response that protects neurons by engulfing damaged mitochondria and thus neutralizing proapoptotic factors to restore neuronal function.^27, 29^

Despite significant experimental evidence that implicates mitochondrial depolarization as a crucial step in the apoptotic pathway,^30^ the molecular mechanism that links brain damage to mitochondrial dysfunction and induction of autophagy machinery, as antiapoptotic mechanism, remains unexamined.

Thus, we first determined the effects of HCb on mitochondrial transmembrane potential (Δ*Ψ*m) that were prepared from axotomized precerebellar neurons (inferior olive and pontine nuclei).

**Figure 1:**
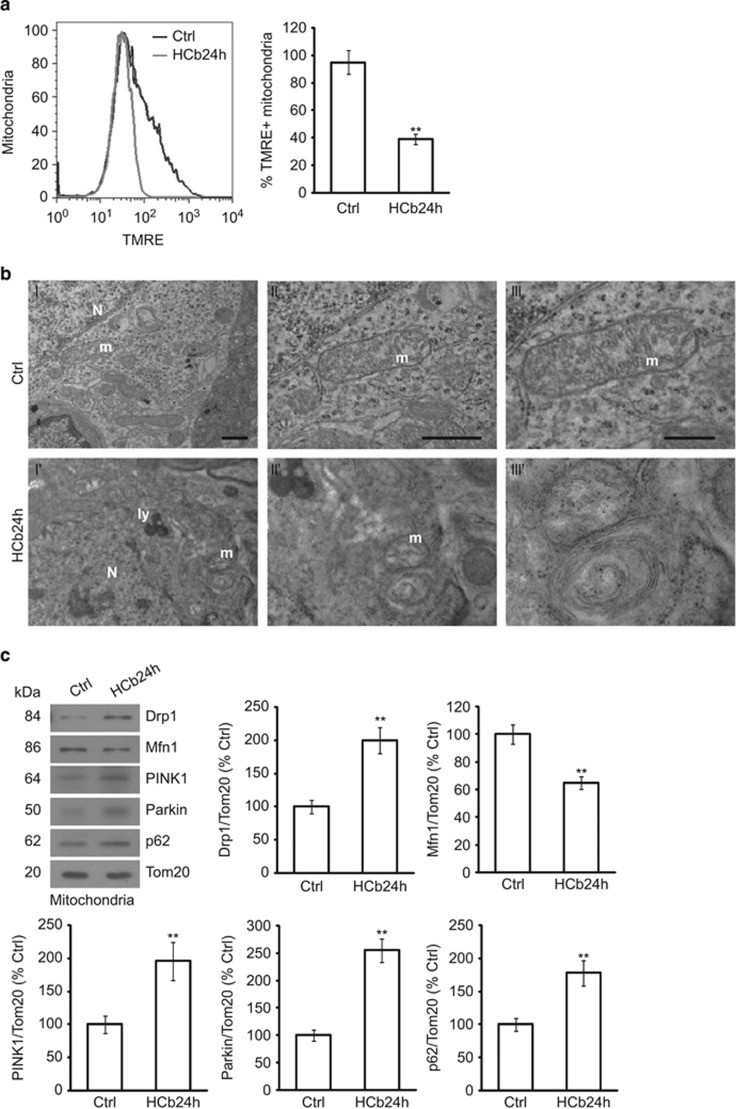
HCb causes mitochondrial membrane depolarization and fragmentation in axotomized precerebellar nuclei. (**a**) Representative cytofluorimetric analysis (left panel) and graph showing quantitative analysis (right panel) of mitochondria isolated from precerebellar nuclei of Ctrl and lesioned mice at 24 h (HCb24h) and stained with tetramethylrhodamine ethyl ester (TMRE). Data are expressed as mean±S.D. (**P<0.01, *n*=4). (**b**) Representative transmission electron microscopy of precerebellar neurons in Ctrl and HCb mice at 24 h (HCb24h). (**II** and **III**) are higher magnification of a mitochondrion (m) shown in **I**. The cristae are well preserved and the electron density of mitochondrial matrix appears as regular. By contrast, the mitochondrion shown in **I**' and enlarged in **II**' and **III**' is profoundly altered, showing disrupted cristae and abnormal matrix. N, nucleus; ly, lysosomal vesicle. Scale bars, 1 *μ*m (**I** and **II**) and 200 nm (**III**). (**c**) Representative immunoblots and densitometric graphs of mitochondrial content of Drp1, Mfn1, PINK1, Parkin and p62 levels (Ctrl is indicated as 100%) in precerebellar nuclei of Ctrl and HCb24h mice. The mitochondrial protein Tom20 was used as loading control. Data are expressed as mean±S.D. (**P<0.01, *n*=5)

By flow cytometric analysis of isolated mitochondria that were stained with the Δ*Ψ*m marker tetramethylrhodamine ethyl ester (TMRE),^31^ mitochondria from precerebellar nuclei of HCb mice at 24 h (HCb24h) were depolarized to a greater extent than in control mice (Ctrl) (*P*<0.01, Ctrl *versus* HCb24h; Figure 1a and Table 1). As Ctrl, all preparations of mitochondria were also stained by TMRE in the presence of trifluorocarbonylcyanide phenylhydrazone (FCCP), resulting in complete loss of membrane potential (Supplementary Figure 1a). This result demonstrates that HCb decreases the Δ*Ψ*m of mitochondria from precerebellar neurons, consistent with our published data, showing that axotomy effects the release of cytochrome *c* from mitochondria (starting 12 h after lesion, before induction of autophagy).

To determine mitochondrial morphology in axotomized neurons, we examined precerebellar neurons of Ctrl and HCb24h mice by transmission electron microscopy. We observed the presence of altered mitochondria in axotomized neurons with abnormal matrix and disrupted cristae (Figure 1b). Notably, we previously demonstrated the presence of double-membraned structures containing mitochondria in axotomized neurons, suggestive of mitochondrial degradation by autophagy.^26^

Mitochondrial damage alters the balance of mitochondrial dynamics – that is, fusion and fission.^8^ Δ*Ψ*m loss is linked to mitochondrial fragmentation^30^ and the E3 ubiquitin ligase Parkin is selectively recruited by PINK1 to damaged and depolarized mitochondria, promoting their degradation by autophagy.^32^ Parkin recruits p62/SQSTM1 (hereafter p62) to depolarized mitochondria by direct ubiquitylation and targets them for mitophagy. In addition, Parkin interacts with and ubiquitinates Mfn1, inducing its degradation and thus preventing the fusion of damaged mitochondria.^19^ Parkin-mediated blockade of mitochondrial fusion is associated with an increase in mitochondrial fission that is sustained by Drp1.^6^ On mitochondrial damage, Drp1 accumulates at mitochondria, where it induces mitochondrial fission. These mitochondrial events might be necessary to selectively isolate depolarized mitochondria for elimination by mitophagy.^20^

To examine these events in axotomized remote neurons after HCb, we purified mitochondria from precerebellar nuclei (Supplementary Figure 1b) 24 h after the lesion and measured the mitochondrial levels of Parkin and Mfn1. Consistent with the mitochondrial membrane depolarization that we observed, we noted significant mitochondrial accumulation of PINK1, Parkin and p62 in HCb24h compared with Ctrl mice; in contrast, the level of Mfn1 was significantly reduced in the same mitochondrial preparation (*P*<0.01, Ctrl *versus* HCb24h; Figure 1c). Moreover, mitochondrial accumulation of Drp1 was significantly greater in HCb24h *versus* Ctrl mice (*P*<0.01, Ctrl *versus* HCb24h; Figure 1c), indicating an imbalance in mitochondrial fusion/fission events, favoring fission.

Notably, cytosolic levels of Drp1 declined after HCb (*P*<0.01 Ctrl *versus* HCb24h; Supplementary Figure 1c), confirming the translocation of Drp1 from the cytosol to the mitochondria. Collectively, these data demonstrated that HCb causes mitochondrial depolarization and perturbs mitochondrial dynamics in axotomized precerebellar neurons.

### Calcineurin mediates mitochondrial fission in the early stages of remote neurodegeneration

**Figure 2:**
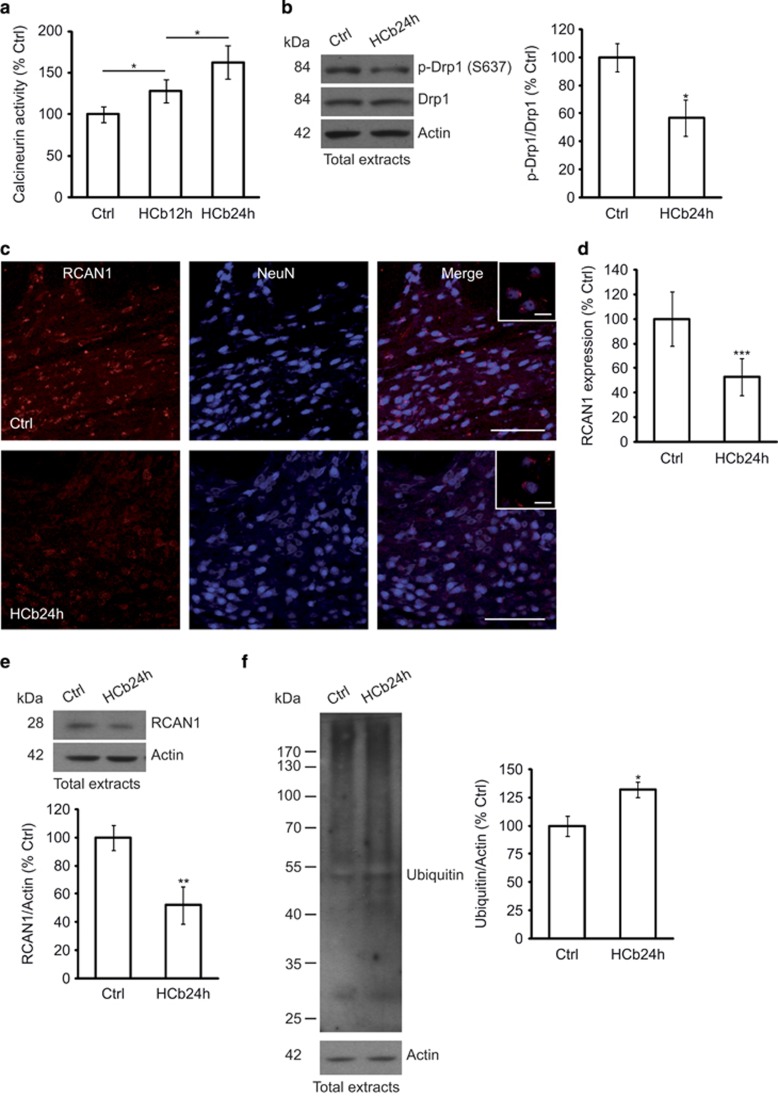
Drp1 translocation to mitochondria in axotomized neurons is associated with increased calcineurin activity, RCAN1 downregulation, and protein ubiquitination. (**a**) Histogram showing calcineurin activity obtained by monitoring ^32^P release from radiolabeled purified RII substrate in axotomized precerebellar nuclei of Ctrl and HCb mice at 12 and 24 h (HCb12h and HCb24h, respectively). Data are expressed as mean (Ctrl percentage)±S.D. (*P<0.05, *n*=5). (**b**) Representative immunoblots and densitometric graphs of p-Drp1(S637)/Drp1 ratio (Ctrl percentage) in precerebellar nuclei of Ctrl and HCb24h mice. Actin was used as loading control. Data are expressed as mean±S.D. (*P<0.05, *n*=5) (**c**) Double-labeled and merged confocal images of RCAN1 (red) and NeuN (blue) in precerebellar nuclei of Ctrl and HCb mice at 24 h. Scale bars, 100 and 20 *μ*m (inset). (**d**) Densitometric graph of RCAN1 levels in Ctrl and HCb24h mice. Data are expressed as mean±S.D. (***P<0.001, *n*=5 mice per group, *N*=150 cells per group). (**e**) Representative immunoblots and densitometric graphs of RCAN1 levels (Ctrl percentage) in precerebellar nuclei of Ctrl and HCb24h mice. Actin was used as loading control. Data are expressed as mean±S.D. (**P<0.01, *n*=5). (**f**) Representative immunoblots and densitometric analysis of proteins extracted from precerebellar nuclei of Ctrl and HCb24h mice probed with anti-multiubiquitin antibody. Actin was used as loading control. Data are expressed as mean±S.D. (*P<0.05, *n*=5). Ctrl is defined as 100%

Drp1 translocation from the cytosol to mitochondria to induce fission is regulated by its serine phosphorylation/dephosphorylation. Specifically, calcineurin-dependent dephosphorylation of Ser637 effects Drp1 translocation to mitochondria, creating a loop in which fragmentation of depolarized mitochondria involves intracellular Ca^2+^ increase, activation of calcineurin, and dephosphorylation and translocation of Drp1.^33^ As axotomy-induced remote degeneration is likely mediated by Ca^2+^ influx and because calcineurin is activated by increased Ca^2+^ levels, we measured calcineurin activity in precerebellar nuclei in response to axotomy at 12 and 24 h after the development of lesion (Table 1). We found that calcineurin activity was significantly increased in axotomized precerebellar nuclei of HCb animals (HCb12h and HCb24h) compared with Ctrls (*P*<0.05, Ctrl *versus* HCb12h and Ctrl *versus* HCb24h; Figure 2a). The specificity of this assay was ensured by the addition of okadaic acid to the reaction buffer at concentrations that inhibit the phosphatase activity of proteins other than calcineurin.

Moreover, the level of p-Drp1 (S637) in total protein extracts from precerebellar nuclei declined significantly in HCb mice at 24 h (*P*<0.05, Ctrl *versus* HCb24h; Figure 2b), suggesting that in this model calcineurin mediates Drp1 dephosphorylation and translocation to mitochondria.

Calcineurin activity is strictly regulated by regulator of calcineurin 1 (RCAN1), which interacts with calcineurin subunit A, inhibiting calcineurin activity.^34, 35^ RCAN1 expression in astrocytes has been shown to have a protective function in brain ischemia/reperfusion injuries.^36^

The pattern of RCAN1 expression in axotomized precerebellar nuclei changed significantly after HCb compared with Ctrl animals (Figure 2c). Further, contrary to a study that reported nearly exclusive glial expression of RCAN1,^36^ RCAN1 was confined solely to neuronal cells in our model (Figure 2c inset) and was absent from glial cells (data not shown). This result, although unexpected, is consistent with previous data on the disparate functions of glia and neurons in mediating the response to damage, depending on the context – that is, acute focal or remote delayed neurodegeneration.^22^ Densitometric analysis of RCAN1 immmunostaining showed that axotomy downregulated RCAN1 expression in precerebellar nuclei compared with the Ctrl (*P*<0.001, Ctrl *versus* HCb24h; Figure 2d). These data were confirmed by western blotting (*P*<0.01, Ctrl *versus* HCb24h; Figure 2e).

The evidence that RCAN1 is ubiquitinated and degraded by chaperone-mediated autophagy and the ubiquitin proteasome pathway,^37^ as well as the experimental findings of enhanced protein ubiquitination in precerebellar nuclei from HCb24h mice (*P*<0.05, Ctrl *versus* HCb24h; Figure 2f), suggest that the increase in calcineurin activity also depends on less inhibition by RCAN1, which can be degraded by the ubiquitin–proteasome system after HCb.

### Rapamycin promotes removal of damaged mitochondria by stimulating fission and autophagy

We have reported that autophagy protects neurons from remote degeneration.^26^ Further, stimulation of autophagy by rapamycin reduces cytochrome *c* release and improves neuronal survival and functional recovery. In contrast, the impairment in autophagy is associated with greater cytochrome *c* release, decreased neuronal survival, and worse functional recovery.^26^ In the current study, we determine whether pharmacological stimulation of autophagy helps eliminate damaged mitochondria and improves mitochondrial function 24 h after HCb. To this end, we treated HCb24h mice with a single dose of rapamycin (2 mg/kg, intraperitoneal (i.p.)) or saline as vehicle Ctrl (Table 1).

**Figure 3:**
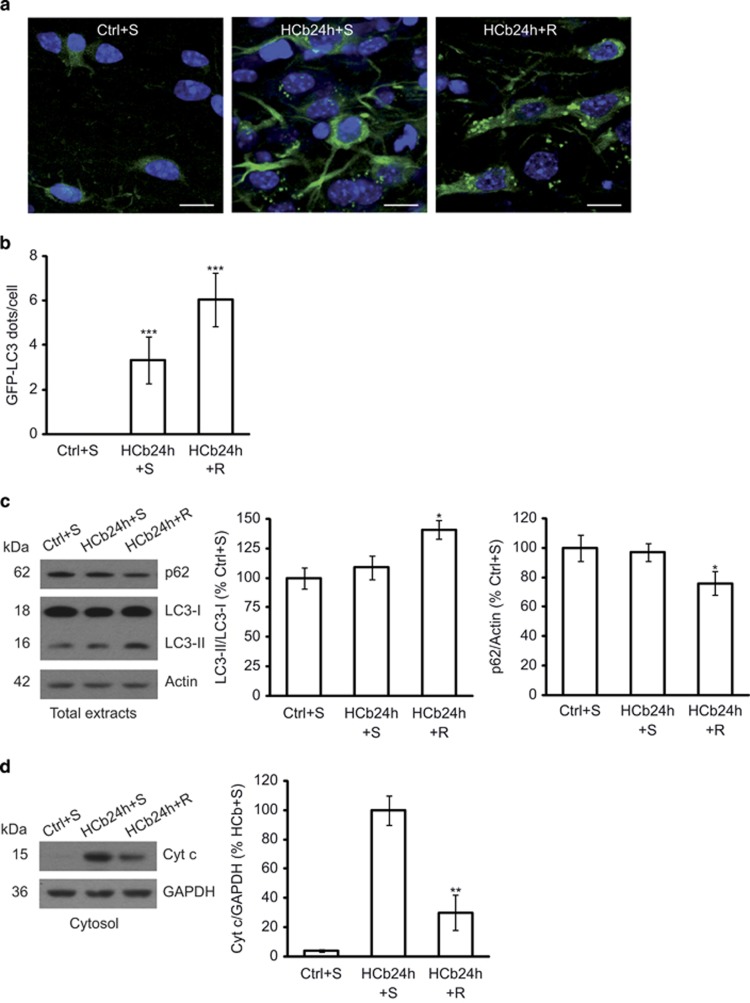
Rapamycin treatment stimulates autophagy in HCb mice at 24 h and reduces cytochrome *c* release into the cytosol. (**a**) Double-labeled and merged confocal images of GFP-LC3 (green) and DAPI (blue) of precerebellar neurons in saline-treated Ctrl GFP-LC3 (Ctrl+S), saline-treated HCb GFP-LC3 (HCb24h+S), and rapamycin-treated HCb GFP-LC3 (HCb24h+R) mice at 24 h. Scale bars, 10 *μ*m. (**b**) Histogram of the number of GFP-LC3 dots per neuron. Data are expressed as mean±S.D. (***P<0.001, *n*=5 mice per group, *N*=150 cells per group). (**c**) Representative immunoblots and densitometric graph of LC3-II/LC3-I ratio and p62 levels in precerebellar nuclei of saline-injected Ctrl (Ctrl+S), saline-injected HCb24h (HCb24h+S), and rapamycin-injected HCb24h (HCb24h+R) mice. Actin was used as loading control. Data are expressed as mean±S.D. (*P<0.05, *n*=5). (**d**) Representative immunoblots and densitometric graph of cytosolic proteins purified from precerebellar nuclei of Ctrl+S, HCb24h+S, and HCb24h+R mice. Cytosolic protein glyceraldehyde 3-phosphate dehydrogenase (GAPDH) was used as loading control. Data are expressed as mean±S.D. (**P<0.01, *n*=5). HCb+S is defined as 100%

To monitor the induction of autophagy after focal brain damage, we performed HCb in transgenic mice that ubiquitously expressed LC3-conjugated green fluorescent protein (GFP)^38^ (Table 1). By confocal analysis, we observed that the axotomy induced GFP-LC3 puncta (Figure 3a), which were not detected in Ctrl mice (as previously demonstrated^26^). This observation reflects the formation of autophagosomes in axotomized precerebellar neurons, notably rapamycin-upregulated GFP-LC3 puncta (Figure 3a), indicating an increase in autophagy. By quantitative analysis, the number of GFP-LC3 puncta in axotomized neurons rose significantly after HCb compared with Ctrl (*P*<0.001, Ctrl+S *versus* HCb24h+S; Figure 3b), and rapamycin increased the number of GFP-LC3 puncta per neuron (*P*<0.001, HCb24h+S *versus* HCb24h+R; Figure 3b). Stimulation of autophagy by rapamycin was confirmed by western blotting (*P*<0.05, HCb24h+S *versus* HCb24h+R; Figure 3c) and, in particular, by the detection of the conversion of non-lipidated LC3 (LC3-I) to lipidated (LC3-II) and by the analysis of p62 expression. LC3-I-to-LC3-II conversion and downregulation of p62 are considered *bona fide* indices of autophagy.^39^

Rapamycin treatment was associated with a significant reduction in cytochrome *c* release (*P*<0.01, HCb24h+S *versus* HCb24h+R; Figure 3d) into the cytosol, as previously reported at various time points (2 and 4 days after HCb).^26^

**Figure 4:**
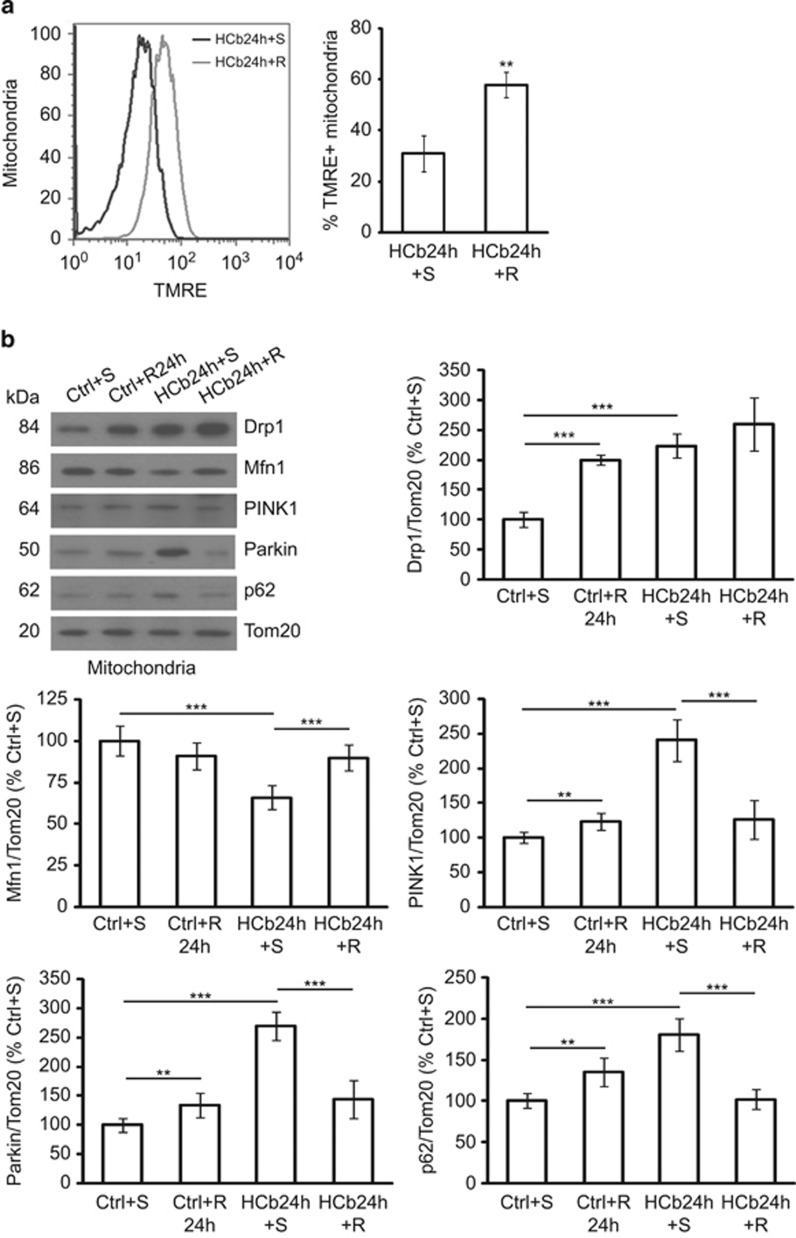
Rapamycin treatment improves mitochondrial function and perturbs mitochondria dynamics. (**a**) Representative cytofluorimetric analysis (left) and quantification graphs (right) of mitochondria isolated from precerebellar nuclei of saline- and rapamycin-treated HCb (HCb24h+S and HCb24h+R, respectively) mice at 24 h and stained with tetramethylrhodamine ethyl ester (TMRE). Data are expressed as mean ±S.D. (**P<0.01, *n*=4). (**b**) Representative immunoblots and densitometric graph of mitochondrial levels of Drp, Mfn1, PINK1, Parkin, and p62 in precerebellar nuclei of saline- and rapamycin-treated Ctrl (Ctrl+S and Ctrl+R24h, respectively) and saline- and rapamycin-treated HCb (HCb24h+S and HCb24h+R, respectively) mice. The mitochondrial protein Tom20 was used as loading control. Data are expressed as mean±S.D. (**P<0.01, ***P<0.001, *n*=7). Ctrl+S is indicated as 100%

Thus, to evaluate the effect of autophagy stimulation on the removal of damaged mitochondria 24 h after HCb, we isolated mitochondria of axotomized precerebellar nuclei from mice that were treated with a single dose of rapamycin (HCb24h+R) or saline solution (HCb24h+S) as Ctrl (Table 1). Mitochondrial TMRE staining revealed that the fraction of polarized mitochondria from rapamycin-treated mice exceeded that of mice that were injected with saline (*P*<0.01, HCb24h+S *versus* HCb24h+R; Figure 4a). As Ctrl, all mitochondrial preparations were also stained with TMRE in the presence of FCCP (Supplementary Figure 1d). This result suggests that the protective effect of rapamycin is attributed to its ability to stimulate the removal of damaged mitochondria. We have demonstrated^26^ a lack of efficacy of rapamycin in mice with impaired autophagic responses (Beclin heterozygous mice), implicating autophagy mechanisms as the principal target of rapamycin-associated neuroprotective effects.

Several lines of evidence suggest that mitochondria influence the autophagic process, and that the stimulation of mitochondrial fission results in autophagy, for example, heavily fused mitochondria are a poor substrate that evades autophagic degradation.^40^

To determine whether rapamycin stimulates mitochondrial Drp1 translocation to promote autophagic degradation of damaged mitochondria after HCb, we purified mitochondria from axotomized precerebellar nuclei of Ctrl and HCb24h mice that were treated with saline or rapamycin, and analysed mitochondrial levels of Drp1 (Figure 4b). In rapamycin-treated Ctrl mice (Ctrl+R24h), we observed an accumulation of Drp1 at mitochondria compared with Ctrl+S mice (*P*<0.001, Ctrl+S *versus* Ctrl+R24h; Figure 4b). Rapamycin also increased Drp1 mitochondrial translocation in HCb24h mice, albeit not statistically different from HCb+S (*P*>0.05, HCb24h+S *versus* HCb24h+R; Figure 4b).

These data suggest that rapamycin, in addition to activating autophagy by inhibiting mammalian target of rapamycin (mTOR), stimulates mitochondrial removal by inducing fission and then favoring mitochondrial elimination by autophagy.

Consistent with the increase in Δ*Ψ*m, rapamycin reduced PINK1 levels, and Parkin and p62 mitochondrial localization after HCb (*P*<0.001, HCb24h+S *versus* HCb24h+R; Figure 4b), concomitant with an increase in Mfn1 levels (*P*<0.001, HCb24h+S *versus* HCb24h+R; Figure 4b).

**Figure 5:**
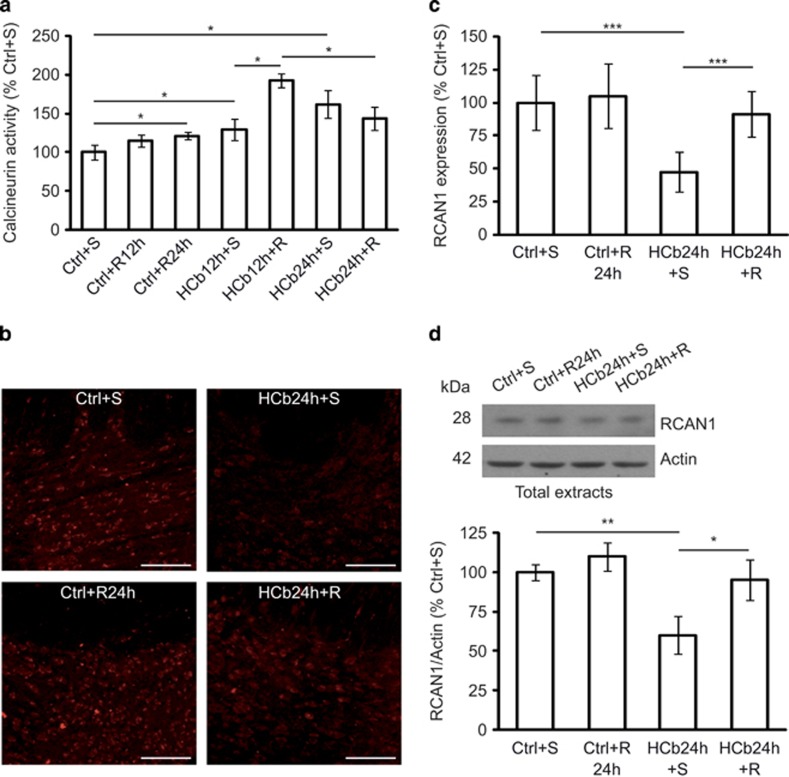
Rapamycin treatment stimulates calcineurin activity. (**a**) Histogram of calcineurin activity obtained by monitoring ^32^P release from radiolabeled purified RII substrate in axotomized precerebellar nuclei of saline- and rapamycin-treated Ctrl (Ctrl+S, Ctrl+R12h, and Ctrl+R24h) and saline- and rapamycin-treated HCb (HCb12h+S, HCb12h+R, HCb24h+S, and HCb24h+R) mice at 12 and 24 h. Data are expressed as mean (Ctrl+S percentage)±S.D. (*P<0.05, *n*=5). (**b**) Confocal images of RCAN1 staining of precerebellar nuclei of Ctrl+S, Ctrl+R, HCb+S, and HCb+R mice at 24 h. (**c**) Densitometric graph of RCAN1 staining in Ctrl+S, Ctrl+R24h, HCb24h+S, and HCb24h+R mice. Data are expressed as mean±S.D. (***P<0.001, *n*=6 mice per group, *N*=180 cells per group). (**d**) Representative immunoblots and densitometric graph of RCAN1 levels (Ctrl+S percentage) in precerebellar nuclei of Ctrl+S, Ctrl+R24h, HCb24h+S, and HCb24h+R mice at 24 h. Actin was used as loading control. Data are expressed as mean ±S.D. (*P<0.05, **P<0.01, *n*=5)

As rapamycin alters Drp1 localization, we examined whether it could also modulate calcineurin activity by analysing calcineurin phosphatase activity in precerebellar nuclei of Ctrl and HCb (at 12 and 24 h) mice that were treated with saline or rapamycin (Table 1). Calcineurin activity rose significantly in Ctrl+R24h compared with Ctrl+S animals (*P*<0.05, Ctrl+S *versus* Ctrl+R24h; Figure 5a), which was evident, albeit not statistically significant, at 12 h (*P*>0.05, Ctrl+S *versus* Ctrl+R12h; Figure 5a).

In contrast, in HCb mice (which have higher levels of calcineurin activity *versus* Ctrl; Figures 2a and 5a), rapamycin further enhanced calcineurin activity only at 12 h after HCb (*P*<0.05, HCb12h+S *versus* HCb12h+R; Figure 5a). At 24 h, rapamycin was not able to increase calcineurin activity but seemed to slightly reduce it (*P*>0.05, HCb24h+S *versus* HCb24h+R; Figure 5a). These data suggest that 12 h after the lesion the rapamycin-induced increase in calcineurin activity stimulates mitochondrial fission, effecting their elimination by mitophagy, whereas at 24 h this effect is no longer detectable. Moreover, we performed this assay in the presence of okadaic acid to exclude the contribution of other phosphatases.

Next, we analyzed RCAN1 expression by confocal analysis (Figures 5b and c) and immunoblotting (Figure 5d). Rapamycin upregulated RCAN1 expression in HCb mice at 24 h (*P*<0.001, HCb24h+S *versus* HCb24h+R (Figure 5c) and *P*<0.05, HCb24h+S *versus* HCb24h+R (Figure 5d)), consistent with the decreased calcineurin activity (although the latter not statistically significant). Rapamycin did not alter RCAN1 levels in Ctrl mice, suggesting that the effects of rapamycin on calcineurin activity are mediated by disparate pathways in unlesioned and lesioned mice.

## Discussion

In this study, we have provided functional evidence that HCb causes mitochondrial depolarization and PINK1 accumulation in axotomized precerebellar neurons; Parkin translocation to depolarized mitochondria and consequent blockade of mitochondrial fusion by Mfn1 degradation; recruitment of p62 at mitochondria; and induction of mitochondrial fission by significant Drp1 accumulation in mitochondria.

Recently, we demonstrated that this paradigm of brain damage enhances autophagy in axotomized precerebellar neurons and, more significantly, that pharmacological stimulation of autophagy improves neurological functions.^26^ In the current study, we examined the functional relationship between two experimental observations – mitochondrial damage and autophagy activity – and characterized the involvement of rapamycin in mitochondrial function.

**Figure 6:**
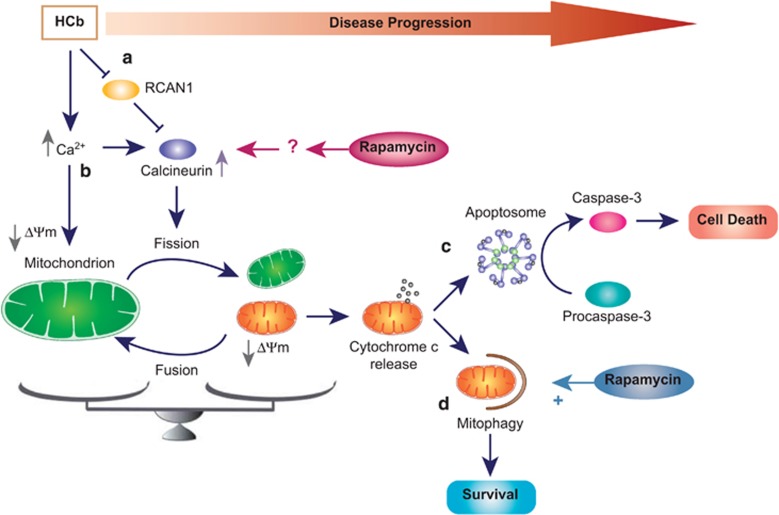
Schematic of the neuroprotective effect of rapamycin in hemicerebelloctomized mice. HCb causes a reduction in levels of the calcineurin inhibitor RCAN1, leading to an increase in calcineurin activity in precerebellar nuclei contralateral to the lesion (**a**). Concomitant with the RCAN1 degradation, the axotomy-induced increase in intracellular Ca^2+^, on the one hand enhances calcineurin activity and on the other hand affects the mitochondrial membrane potential (Δ*Ψ*m) (**b**). Calcineurin activity promotes mitochondrial fission inducing Drp1 translocation (see text). Damaged mitochondria release cytochrome *c* into the cytosol, activating the apoptotic pathway, leading to cell death (in absence of mitophagy) (**c**). Prompt activation of the autophagic pathway might help remove damaged organelles by mitophagy, promoting neuronal survival (**d**). In this model, rapamycin treatment has two functions: (1) stimulate autophagy, leading to removal of damaged mitochondria; and (2) enhance mitochondrial fission to effect their elimination by mitophagy

Greater intracellular calcium levels are considered to be a key element in neuronal death that is induced in various conditions, such as cerebral ischemia and head trauma.^41, 42^ Chronic increases in the intracellular calcium (Figure 6), under neuronal stress conditions, affect the mitochondrial membrane potential (Δ*Ψ*m), causing mitochondrial dysfunction, characterized by bioenergetic failure and release of proapoptotic factors, such as cytochrome *c*, into the cytosol.^43, 44, 45^ Thus, to prevent neuronal apoptosis it is necessary to remove the dysfunctional mitochondria preventing fusion, increasing fission, and selectively removing damaged mitochondria.^46^

In this study, we demonstrated that after damage, mitochondrial fusion is blocked by Parkin-mediated degradation of Mfn1. Concurrently, intracellular calcium overload activates (Figure 6) signal transduction pathways that are mediated by activation of calcium-dependent enzymes, including the protein phosphatase calcineurin, as we have shown. Once activated, calcineurin dephosphorylates cytosolic Drp1 (at Ser637), which translocates to mitochondria and prompts mitochondrial fission. In this process, autophagy acts as a catabolic process in which fragmented and depolarized mitochondria are degraded. Accordingly, we observed that rapamycin-induced stimulation of autophagy led to partial rescue of neuronal viability and significant improvement in neurological function.^26^

We also found that rapamycin injection, in addition to stimulating autophagy, increases calcineurin activity in unlesioned and HCb (at 12 h after the lesion) mice, promoting Drp1 mitochondrial accumulation and fission, and faster degradation of damaged mitochondria by autophagy.

What is the relationship between axotomy, calcineurin activity, autophagy, and rapamycin?

We observed that HCb (Figure 6) downregulates RCAN1 (calcineurin inhibitor) levels *versus* Ctrl in axotomized neurons, suggesting that the rise in calcineurin activity in lesioned mice depends on the reduction in RCAN1 expression in addition to calcium overload. Thus, the beneficial effects of rapamycin might result from increased degradation of RCAN1 in the very early stages after HCb, enhancing calcineurin activity and mitochondrial fission. In contrast, 24 h after HCb, when the clearance of damaged mitochondria is ongoing, the RCAN1 expression and calcineurin activity return progressively to physiological levels.

Notably, rapamycin treatment in unlesioned mice had no effects on RCAN1 expression, despite increasing calcineurin activity and Drp1 mitochondrial translocation. Based on these findings, we do not exclude the possibility that rapamycin has a direct mTOR-dependent effect on calcineurin, unrelated to RCAN1 expression, as reported for the protein phosphatase 2A phosphatase.^47^

Collectively, our data demonstrate a strong relationship *in vivo* between mitochondrial dynamics and autophagy as the molecular mechanism for degrading damaged mitochondria (Figure 6). Moreover, we identified rapamycin as a pharmacological tool that can be used to promote mitochondrial fission – a necessary step in dismantling damaged mitochondria and preventing uncontrolled mitochondria-dependent caspase-3 activation and neuronal apoptosis.^48^

Our results are consistent with the findings of other studies,^49, 50^ demonstrating that the clearance of damage mitochondria reduces mitochondria-dependent apoptosis and subsequently prevents neuronal death.

In conclusion, we have shown that in remote brain damage autophagy removes damaged mitochondria that would have otherwise induced neuronal death, thus protecting against secondary brain damage. Thus, based on the effects of autophagy in acute brain pathologies, rescuing or reducing brain damage by early pharmacological degradation of dysfunctional mitochondria should be considered.

## Materials and Methods

### Animals

Experiments were performed using adult mice (weight 20–25 g; Table 1). C57BL6 mice (10–12 weeks of age) were obtained from Harlan Italy (San Pietro Al Natisone, Italy) and GFP-LC3 transgenic mice were provided by Dr N Mizushima. The experimental protocol was approved by the Italian Ministry of Health per the guidelines of the European Communities Council Directive, 24 November 1986 (86/609/ EEC), for the care and use of laboratory animals. All efforts were made to minimize the number of animals used and their suffering.

### Surgery and drug treatment

Cerebellar lesions were induced by performing a right HCb, as previously described.^26^ For surgical procedures, the mice were deeply anesthetized by i.p. injections of xylazine (Rompun, 10 mg/ml; Bayer, Leverkusen, Germany) and tiletamine e zolazepam (Zoletil 100, 50 mg/ml; Virbac, Carros, France), and were positioned in a stereotaxic apparatus. An incision was made in the skin on the skull and the occipital bone was drilled and removed. Subsequently, the dura was incised to expose the cerebellum and the right cerebellar hemisphere was removed by suction. The wound was sutured and the animals were returned to their cages. For the Ctrl group, surgery was interrupted after the dura madre lesion was made, and after suturing the animals were returned to their cages. Rapamycin (Alexis Biochemicals, San Diego, CA, USA; 380-004-M001) was dissolved in DMSO (25 mg/ml) and injected 2 mg/kg i.p.

### Mitochondria isolation and TMRE staining

Isolation of mitochondria from mouse brain tissue was carried out by revising the protocol previously described by Frezza *et al.*^51^ Mouse precerebellar nuclei (two inferior olive (IO) and two pontine nuclei (Pn) for each sample) were rinsed in ice-cold IBc (250 mM sucrose, 10 mM Tris-MOPS, pH 7.4, 1 mM EGTA-Tris, pH 7.4) and homogenated by 40 strokes with a glass/Teflon pestle. Afterwards, the homogenate was centrifuged at 600 × *g* for 10 min and the supernatant was collected and centrifuged at 7000 × *g* for 10 min. The pellet was gently resuspended in ice-cold IBc and washed at 7000 × *g* for 10 min. The supernatant was discarded and the pellet, containing mitochondria, was gently resuspended in the small amount of residual buffer. Bradford method was used to evaluate the mitochondria concentration and the proteins (50 *μ*g) were resuspended in equal quantity of EBm (250 mM sucrose, 10 mM Tris-HCl, pH 7.4, 5 mM MgCl_2_, 20 *μ*M EGTA-Tris, pH 7.4, 2 mM Pi) supplemented with 5 mM glutamate, 2.5 mM malate and 75 nM TMRE perchlorate dye. Next, the samples were incubated for 10 min at 37 °C in the dark and acquired to cytometer. For each sample, 100 000 events were acquired to FacsCanto (BD Biosciences, San Jose, CA, USA). In addition, all samples were treated with 60–100 nM of FCCP to get the complete loss of membrane potential as Ctrl.

### Electron microscopy

Animals were transcardially perfused with a calcium-free Ringer's variant (pH 7.3), followed by 2% freshly depolymerized paraformaldehyde and 1% glutaraldehyde in 0.12 M phosphate buffer (PB), pH 7.4. Brains were dissected out and cut on a vibratome in the coronal plane, to obtain 100-mm-thick sections, which were collected in PB, pH 7.4. Slices were post fixed in 1% osmium tetroxide in PB for 1 h at 4 °C, in the dark, then gradually dehydrated in ethanol. All the steps of the above procedure were performed at 4 °C. Sections were infiltrated with graded mixtures of propylene oxide (Sigma, Milan, Italy; 45345) and Epon812 (TAAB Laboratories, Berkshire, UK; T031), then flat-embedded in the same resin, allowing specimens to polymerize at 60 °C, for 3 days. Selected areas were then remounted on Epon blanks and sectioned by a Reichert Ultracut S ultramicrotome (Leica Microsystems, Wetzlar, Germany), to obtain ultra-thin sections (60–70 mm), which were collected on nickel grids. These were briefly contrasted with 1% uranyl acetate and observed in a Philips CM120 electron microscope (Philips, Amsterdam, The Netherlands), equipped with a Philips Megaview III videocamera (Philips). Images were electronically captured by AnalySys 2.0 software (Leica Microsystem, Milan, Italy).

### Mitochondria purification from brain tissue

Isolation of mitochondria was performed as previuosly described,^52^ with little modifications. Precerebellar nuclei (three IO and three Pn for each sample) were homogenized in 0.5 ml ice-cold Buffer A (320 mM sucrose, 1 mM EDTA, 50 mM Tris-HCl, pH 7.4, 1 mM DTT, 1 mM PMSF, protease inhibitor cocktail) by 30 strokes with a glass Pyrex micro homogenizer. The homogenate was centrifuged at 1000 × *g* for 10 min and the resulting supernatant was centrifuged at 10 000 × *g* for 20 min, to obtain the mitochondrial pellet and the supernatant. The supernatant was centrifuged at 100 000 × *g* for 1 h (SW 60Ti rotor, Beckman Coulter, Milan, Italy) to generate the cytosolic fraction. The mitochondria-containing pellet was washed three times with 0.5 ml of Buffer B (250 mM sucrose, 1 mM EGTA, 10 mM Tris-HCl, pH 7.4) by centrifugation for 10 min at 10 000 × *g*. Crude mitochondria were resuspended in 0.5 ml of Buffer B and further purified by layering the preparation on top of a gradient consisting of 1.1 ml of 2.5 M sucrose, 3.28 ml of Percoll solution, and 12.2 ml of TE Buffer (10 mM Tris-HCl, pH 7.4, 1 mM EDTA). Samples were centrifuged at 60 000 × *g* for 50 min (SW 41Ti rotor, Beckman Coulter). A dense band recovered from approximately two-thirds down the tube, corresponding to purified mitochondria, was collected, diluted with Buffer B, and centrifuged at 10 000 × *g* for 20 min. The resulting pellet was washed three times with 1 ml of Buffer B by centrifugation at 10 000 × *g* for 15 min.

### Protein extraction

Mitochondrial proteins were extracted by resuspending the pellet in a small volume of RIPA Buffer (50 mM Tris-HCl, pH 7.4, 1% Triton X-100, 0.25% sodium deoxycholate, 150 mM NaCl, 5 mM MgCl_2_, 1 mM EDTA, 0.1% SDS, protease inhibitors cocktail), sonicated and incubated on ice for 20 min. The samples were centrifuged at 11 500 × *g* for 10 min and the protein concentration of resulting supernatant was determined by Bradford method. Cytosolic proteins were precipitated by incubation with eight volume of acetone at −20 °C overnight. The pellet obtained by 15 min centrifugation et 3000 × *g* was resuspended in RIPA buffer and sonicated before the determination of protein concentration.

Total extracts were obtained by homogenization of precerebellar nuclei in Lysis Buffer (320 mM sucrose, 50 mM NaCl, 50 mM Tris-HCl, pH 7.4, 1% Triton X-100, 1 mM Na_3_VO_4_, 5 mM 2-glycerophosphate, 5 mM NaF, 20 mM 2-chloroacetamide, proteases inhibitors cocktail), incubated on ice for 30 min and centrifuged at 13 000 × *g* for 10 min. The total protein content of resulting supernatant was determined by Bradford assay.

### Calcineurin activity assay

Calcineurin activity was assayed by monitoring ^32^P release from radiolabeled purified RII substrate peptide.^53, 54^ The precerebellar nuclei were homogenized in buffer A (50 mM Tris-HCl, pH 7.4, 1 mM EDTA, 1 mM CaCl_2_, 1 mM PMSF, 1 mM DTT), subjected to repeated freezing in liquid N_2_ and thawing at 37 °C, and centrifuged at 17 000 × *g* for 10 min. Equal amount of total proteins (20 *μ*g) from the clear supernatant was diluted with buffer B (100 mM Tris-HCl, pH 7.4, 1 mM MnCl_2_, 0.1 mM CaCl_2_, 0.5 mg/ml BSA, 1 mM DTT) and immediately processed for enzyme assay. Addition of 1 mM NiSO_4_ in buffer B was used to obtain maximal activation of the enzyme together with 200 nM okadaic acid at a final concentration known to inhibit phosphatase activities other than calcineurin. Addition of MgCl_2_ was avoided to prevent activation of protein phosphatase 2C.

### Immunofluorescence

Ctrl and HCb animals were perfused transcardially with saline followed by 4% paraformaldehyde in PB (0.1 M, pH 7.4) under renewed general anesthesia induced by i.p. injections of sodium pentobarbital (60 mg/kg). Each brain was removed from the skull, post-fixed in the same fixative for 2 h, and then transferred to 30% sucrose in PB at 4 °C until it sank. Brainstem and cerebellum were cut into sections using a freezing microtome and collected in PB.

Sections were incubated overnight with primary antibodies including mouse anti-neuronal nuclei (NeuN; 1 : 200; Millipore, Darmstadt, Germany; MAB-377), rabbit anti-DSCR1 (RCAN1; 1 : 200, Santa Cruz, Dallas, TX, USA; sc-66864) prepared in PB containing 0.3% Triton X-100. Each incubation step was followed by three 5-min rinses in PB. Afterwards, sections were incubated 2 h at RT with secondary antibodies including Alexa Fluor 555 donkey anti-rabbit IgG (1 : 200; Molecular Probes; Eugene, OR, USA; A31572), Alexa Fluor 488 donkey anti-mouse IgG (1 : 200; Molecular Probes, 31571). Sections were examined under a confocal laser-scanning microscope (Zeiss LSM700).

### Immunoblotting and antibodies

Equal amounts of proteins were applied to SDS-PAGE and electroblotted on a PVDF membrane. Immunoblotting analysis was performed using a chemiluminescence detection kit. The relative levels of immunoreactivity were determined by densitometry using the free software ImageJ (National Institutes of Health, Bethesda, MD, USA).

Primary antibodies used were as folllows: Actin (1 : 20 000; Sigma-Aldrich, St. Louis, MO, USA; A5060), glyceraldehyde 3-phosphate dehydrogenase (1 : 10 000; Calbiochem, Darmstadt, Germany; CB1001), Tom20 (1 : 1000; Santa Cruz, sc-11415), MnSOD (1 : 2000; Enzo Life Sciences, Lausen, Switzerland; ADI-SOD-110), DLP1 (Drp1, 1 : 2000; BD Transduction Laboratories, San Jose, CA, USA; 611112), phospho-Drp1 Ser637 (1 : 1000; Cell Signaling, Danvers, MA, USA; 4867), Mfn1 (1 : 1000; Santa Cruz, sc-50330), parkin (1 : 500; Santa Cruz, sc-32282), LC3 (1 : 250; NanoTools, Teningen, Germany; 0231-100/LC3-5F10), p62/SQSTM1 (1 : 1000; MBL, Woburn, MA, USA; PM045), PINK1 (1 : 1000; Sigma-Aldrich, P0051), DSCR1 (RCAN1, 1 : 1000; Santa Cruz, sc-66864), cytochrome c (1 : 1000; BD Pharmingen, San Jose, CA, USA; 556433), multi ubiquitin (1 : 1000; MBL, D058-3).

Secondary antibodies used were as folllows: goat anti-mouse IgG (1 : 3000; Bio-Rad 170–6516), goat anti-rabbit IgG (1 : 3000; Bio-Rad 170–6515).

## Figures and Tables

**Table 1:** Lesion, treatments, and survival time in the different experimental groups

**Group**	**HCb**	**Treatment**	**Strain**	***N***	**Survival time (h)**
Ctrl		/	C57Bl/6	29	24
HCb12h	X	/	C57Bl/6	5	12
HCb24h	X	/	C57Bl/6	38	24
Ctrl+S		20 *μ*l Saline, i.p.	C57Bl/6	25	24
Ctrl+R12h		Rapamycin 2 mg/kg, i.p.	C57Bl/6	5	12
HCb12h+S	X	20 *μ*l Saline, i.p.	C57Bl/6	5	12
HCb12h+R	X	Rapamycin 2 mg/kg, i.p.	C57Bl/6	5	12
Ctrl+R24h		Rapamycin 2 mg/kg, i.p.	C57Bl/6	25	24
HCb24h+S	X	20 *μ*l saline, i.p.	C57Bl/6	38	24
HCb24h+R	X	Rapamycin 2 mg/kg, i.p.	C57Bl/6	38	24
GFP-LC3 Ctrl+S		20 *μ*l Saline, i.p.	C57Bl/6 GFP-LC3	5	24
GFP-LC3 HCb24h+S	X	20 *μ*l Saline, i.p.	C57Bl/6 GFP-LC3	5	24
GFP-LC3 HCb24h+R	X	Rapamycin 2 mg/kg, i.p.	C57Bl/6 GFP-LC3	5	24

Abbreviations: Ctrl, control; HCb, hemicerebellectomy; i.p., intraperitoneal.

## Abbreviations

OMM: outer mitochondrial membrane
Mfn1: mitofusin 1
Mfn2: mitofusin 2
Drp1: dynamin-related protein 1
Fis1: fission protein 1
PINK1: PTEN-induced putative kinase 1
PD: Parkinson's disease
CNS: central nervous system
HCb: hemicerebellectomy
Δ*Ψ*m: mitochondrial transmembrane potential
TMRE: tetramethylrhodamine ethyl ester
Ctrl: control
FCCP: trifluorocarbonylcyanide phenylhydrazone
RCAN1: regulator of calcineurin 1
i.p.: intraperitoneal
GFP: green fluorescent protein
mTOR: mammalian target of rapamycin
