# Supplementary figures and images for: Acute focal brain damage alters mitochondrial dynamics and autophagy in axotomized neurons
Source: Cell Death Dis. 2014 Nov 27;5(11):e1545–. doi: 10.1038/cddis.2014.511 (PMC4260762; doi:10.1038/cddis.2014.511)

Supplementary Figure 1

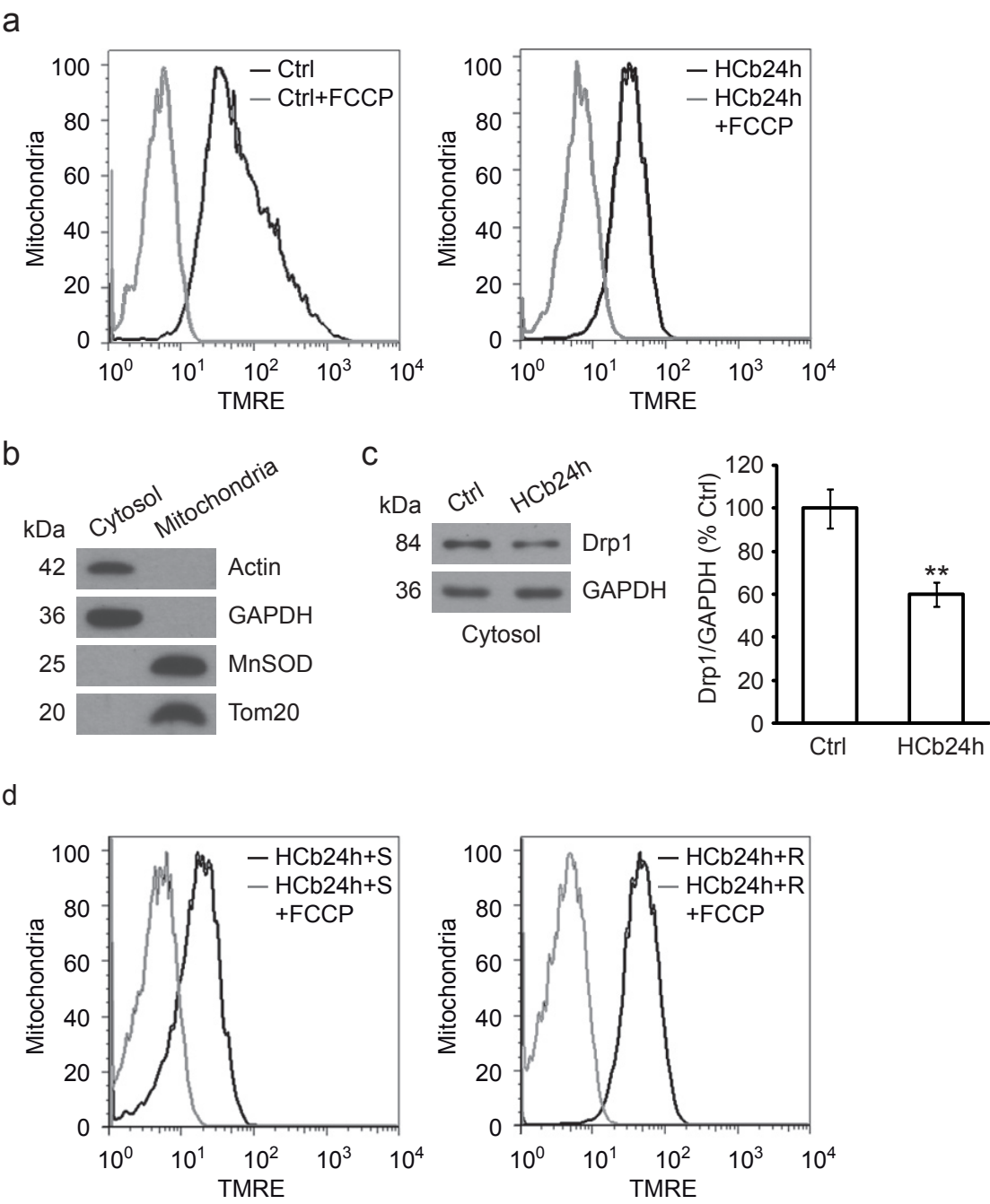

Supplement: Supplementary Figure 1 [file cddis2014511x1.pdf]
