# Supplementary material for: Acute focal brain damage alters mitochondrial dynamics and autophagy in axotomized neurons
Source: Cell Death Dis. 2014 Nov 27;5(11):e1545–. doi: 10.1038/cddis.2014.511 (PMC4260762; doi:10.1038/cddis.2014.511)
Supplement: Supplementary Figure 1 Legends [file cddis2014511x2.doc]

**Supplementary Legend to figures**

**Supplementary Figure 1** **(a)** Representative tetramethylrhodamine ethyl ester (TMRE) staining of mitochondria isolated from precerebellar nuclei of Ctrl (left panel) and HCb24h (right panel) mice in the absence and presence of FCCP (+FCCP). **(b)** Representative immunoblots of mitochondrial and cytosolic proteins purified from precerebellar nuclei demonstrating the purity of the subcellular fractionation. Actin and GAPDH were used as cytosolic markers, and MnSOD and Tom20 were used as mitochondrial markers. **(c)** Representative immunoblots and densitometric graphs of cytosolic content of Drp1 (Ctrl percentage) in precerebellar nuclei of Ctrl and HCb24h mice. Cytosolic protein GAPDH was used as loading control. Data are expressed as mean ± s.d. (** p<0.01, n=5). **(d)** Representative tetramethylrhodamine ethyl ester (TMRE) staining of mitochondria isolated from precerebellar nuclei of saline- (HCb24h+S) and rapamycin-injected HCb (HCb24h+R) mice at 24 h (left and right panel, respectively) in the absence and presence of FCCP (+FCCP).
